# Feasibility of CT quantification of intratumoural ^166^Ho-microspheres

**DOI:** 10.1186/s41747-020-00157-2

**Published:** 2020-05-11

**Authors:** R. C. Bakker, R. Bastiaannet, S. A. van Nimwegen, A. D. Barten-van Rijbroek, R. J. J. Van Es, A. J. W. P. Rosenberg, H. W. A. M. de Jong, M. G. E. H. Lam, J. F. W. Nijsen

**Affiliations:** 1 Department of Radiology and Nuclear Medicine, University Medical Center Utrecht, Utrecht, The Netherlands; 2 Department of Oral and Maxillofacial Surgery, University Medical Center Utrecht, Utrecht, The Netherlands; 3 Department of Clinical Sciences of Companion Animals, Faculty of Veterinary Medicine, Utrecht University, Utrecht, The Netherlands; 4 Department of Head and Neck Surgical Oncology, UMC Utrecht Cancer Center, Utrecht, The Netherlands; 5 Department of Radiology, Nuclear Medicine and Anatomy, Radboudumc, Nijmegen, The Netherlands

**Keywords:** Brachytherapy, Head and neck neoplasms, Humans, Radioisotopes, Tomography (x-ray computed)

## Abstract

**Background:**

Microspheres loaded with radioactive ^166^Ho (^166^Ho-MS) are novel particles for radioembolisation and intratumoural treatment. Because of the limited penetration of β radiation, quantitative imaging of microsphere distribution is crucial for optimal intratumoural treatment. Computed tomography (CT) may provide high-resolution and fast imaging of the distribution of these microspheres, with lower costs and widespread availability in comparison with current standard single-photon emission tomography (SPECT) and magnetic resonance imaging. This phantom study investigated the feasibility of CT quantification of ^166^Ho-MS.

**Methods:**

CT quantification was performed on a phantom with various concentrations of HoCl and Ho-MS to investigate the CT sensitivity and calibrate the CT recovery. ^166^Ho-MS were injected into *ex vivo* tissues, in VX-2 cancer-bearing rabbits, and in patients with head-neck cancer, to demonstrate sensitivity and clinical visibility. The amount of Ho-MS was determined by CT scanning, using a density-based threshold method and compared with a validated ^166^Ho SPECT quantification method.

**Results:**

In the phantom, a near perfect linearity (least squares *R*^2^ > 0.99) between HU values and concentration of ^166^Ho was found. *Ex vivo* tissue experiments showed an excellent correlation (*r* = 0.99, *p* < 0.01) between the dose calibrator, SPECT, and CT imaging. CT recovery was on average 86.4% *ex vivo*, 76.0% in rabbits, and 99.1% in humans.

**Conclusion:**

This study showed that CT-based quantification of Ho microspheres is feasible and is a high-resolution alternative to SPECT-based determination of their local distribution.

## Key points

- Microspheres loaded with radioactive ^166^Ho (166Ho-MS) are novel particles for radioembolisation and intratumoural treatment.
- A phantom study showed a near perfect linearity between HU values on computed tomography (CT) images and concentration of ^166^Ho.
- CT recovery was on average 86.4% *ex vivo*, 76.0% in rabbits, and 99.1% in humans.
- CT can provide fast, high-resolution, and three-dimensional information on the distribution of ^166^Ho-MS for future anti-cancer therapy.

## Background

A relatively new oncological treatment option is selective internal radiation therapy using small radioactive particles, also called radioembolisation. Radioactive holmium-166 (^166^Ho)-loaded (poly L-lactic acid) microspheres (^166^Ho-MS) were developed for radioembolisation of liver tumours through intra-arterial injection [1]. These ^166^Ho-MS have a mean diameter of 30 μm and emit high-energy β particles for tumour treatment and also γ radiation, which allows for quantitative single-photon emission computed tomography (SPECT) imaging [2, 3]. In addition, Ho is paramagnetic, which potentially allows for using magnetic resonance imaging for its recovery (MRI). Both imaging modalities are used in radioembolisation to investigate the ^166^Ho-MS distribution [1, 3, 4].

The intratumoural injection of radioactive microparticles might be beneficial for selected patients with tumours that can be reached with a needle [5–8]. This kind of “micro-brachytherapy” might have advantages, including minimally invasive handling, outpatient treatment, and potentially improved (progression-free) survival and quality of life [5, 8, 9]. In patients with unresectable hepatocellular carcinoma, 12 weeks after intratumoural injection of radioactive ^32^P-microparticles treatment, all tumours showed response with a complete remission in 25% of the lesions [7]. The first experience with ^166^Ho-MS in recurrent head and neck cancer patients showed the minimally invasive character and relative safety of this treatment. However, it also revealed challenges in obtaining a homogeneous absorbed dose and sufficient tumour coverage [8].

Intratumoural treatment with ^166^Ho-MS has also been conducted in several animal studies [6, 10–12]. In 13 companion animals (cats) with spontaneous inoperable oral squamous cell carcinomas, a local response rate of 55% was obtained with minimal side effects [6]. The distribution of ^166^Ho-MS in these animals was imaged with planar scintigraphy in the absence of SPECT or MRI. During the follow-up, computed tomography (CT) imaging clearly showed the distribution of these microspheres [6].

Thus, CT imaging could offer a quicker and higher-resolution feedback on the three-dimensional (3D) distribution of ^166^Ho-MS compared to SPECT and MRI, apart from being more widely available and at relatively lower cost. CT scanning may be used to confirm that a homogeneous absorbed dose and sufficient tumour coverage was achieved and eventually facilitate an image-guided treatment approach. The purpose of this study was to assess the feasibility of ^166^Ho-MS quantification using CT.

## Methods

### ^166^Ho-MS preparation

Non-radioactive ^165^Ho-MS were prepared as previously described [13]. Neutron activation of the Ho-loaded microspheres was performed via the ^165^Ho (n, ɣ) ^166^Ho reaction in a nuclear reactor with a nominal thermal neutron flux of 5 × 10^12^ cm^−2^ s^−1^ (Delft University of Technology). Therefore, vials with known amounts of ^165^Ho-MS were irradiated up to 2 h. After neutron activation, ^166^Ho-MS emit β radiation for tumour ablation (*E*_β, max_ = 1.84 MeV) and γ radiation for imaging (*E*_γ_ 80.6 keV, 6.71%) and has a half-life time of 26.8 h. In the *in vitro*, *ex vivo* and rabbit experiments, a batch of 18.7% weight by weight (Quiremspheres) was used, and in the human patients, a batch of ^166^Ho-MS with a Ho content of 17.6% weight by weight was used, corresponding to approximately 1 mg of Ho per 5.5 mg ^166^Ho-MS.

### Phantom calibration for CT imaging

To quantify ^165^Ho-MS on CT imaging, series of HoCl_3_ and ^165^Ho-MS concentrations were made to create a calibration curve. A serial dilution (*n* = 10) of HoCl_3_ hexahydrate (HoCl_3_ • 6 H_2_O) (Sigma-Aldrich Chemie N.V., Zwijndrecht, The Netherlands) was made in sterile demineralised water (Versylene, Fresenius Kabi, B.V., Huis Ter Heide, The Netherlands) to obtain a homogeneous solution (range 0.0008–0.06 mmol/mL Ho). Therefore, a stock solution was prepared of 0.06 mmol/mL Ho, mixed for 5 min, and diluted by adding demineralised water. The concentration of the solutions was corrected for the mass percentage of Ho of the HoCl_3_ (43.47%).

Two batches of 1,200 mg ^165^HoMS, mass percentage 17.6% and 18.7% [14], were suspended in the injection solution containing 116 mmol phosphate (pH 7.2)-buffered saline with polyoxyethylene-polyoxypropylene block copolymer (Pluronic F-68**,** Sigma-Aldrich Chemie N.V., Zwijndrecht, The Netherlands) 2% weight per volume solution, and then diluted to ten concentrations (range 0.25–20.0 mg/mL^165^HoMS or 0.0016–0.12 mmol/mL Ho) of ^165^HoMS. The ^165^HoMS solutions were subsequently mixed 1:1 with a 2% agar (MP Agar, Roche Diagnostics, Almere, The Netherlands) solution to prevent settling due to the weight of these Ho microspheres (1.4 g/mL). Therefore, the agar powder was dissolved in sterile water (Versylene, Fresenius Kabi, B.V., Huis Ter Heide, The Netherlands) and heated to 90 °C for 10 min, resulting in a transparent fluid. Subsequently, a 5 mL Eppendorf tube was filled with 5 mL of the homogeneously distributed Ho microspheres in agar solution in a series (*n* = 10) with a concentration ranging from 0.125 to 10.0 mg/mL Ho. Once cooled to room temperature in approximately 5 min of continuous rotation to prevent settling, the agar became solid.

### *Ex vivo* and *in vivo* administration of ^166^HoMS

Radioactive ^166^Ho-MS with a known specific activity (Bq/mg) were suspended in a solution of 2% weight by volume Pluronic® F-68 (Sigma-Aldrich Chemie N.V., Zwijndrecht, The Netherlands) in a 116-mmol phosphate (pH 7.2) buffer by gentle agitation and repeatedly drawing up and down in a 1 mL Luer-lock syringe (Becton Dickinson S.A., Madrid, Spain). The injections of the ^166^HoMS were performed with a 1-mL Luer-lock syringe through a 21G × 1½″ (0.8 × 40 mm) hypodermic needle (Becton Dickinson S.A., Madrid, Spain). The activity of the ^166^HoMS inside the syringes was measured using a calibrated dose calibrator (VDC-404; Veenstra Instruments, Joure, The Netherlands). The injection procedures were performed by the intratumoural Ho research team. *Ex vivo* and *in vivo* (laboratory animals) injections were performed by RCB, FN, and BvN. In human patients, injections were performed under ultrasound guidance by ML (nuclear physician) assisted by other members of the team.

### *Ex vivo* CT quantification of ^166^Ho-MS

The feasibility of CT Ho quantification was evaluated in five samples of *ex vivo* chicken muscle tissue. Syringes with approximately 0.2 mL of ^166^Ho-MS suspension with increasing activity ranging from 15 to 81 MBq (corresponding to 3.4 to 18.4 mg Ho) were injected into the tissue samples. The actually injected amount of Ho (mg) was determined by the injected activity. To determine the injected activity, the syringes (before and after injection), the gauze with potential injection channel leakage, and the tissue sample were measured in the dose calibrator. Based on these measurements, the known specific activity of the ^166^Ho-MS and the weight and mass percentage of the ^166^Ho-MS as well as the injected amount (mg) of Ho was calculated.

### Laboratory animals

All experiments were performed in agreement with “The Netherlands Experiments on Animals Act” (1977) and “The European Convention for the Protection of Vertebrate Animals used for Experimental Purposes” (Strasbourg, 18.III.1986). Approval was obtained from the Utrecht University Animal Experiments Committee (DEC 2011.III.08.080).

The VX-2 tumour model in New Zealand white rabbits was previously described [15]. In short, in one animal, the donor rabbit, a tumour was implanted by injection of a suspension of approximately 4.0 × 10^7^ VX-2 carcinoma cells subcutaneously into both flanks. Single tumours were induced in each of five rabbits by harvesting the tumour from the donor rabbit. A subcutaneous injection of 3 ± 1 mm^3^ viable fragments of VX-2 carcinoma with 0.1–0.3 mL phosphate-buffered saline was performed into the flank of five adult female New Zealand White rabbits weighing 3–4 kg. All tumour implantations and treatments were performed under analgesia with carprofen 4 mg/kg. During the animal experiments, sedation and analgesia were achieved with a mixture of 0.125 mg/kg dexdomitor and 15 mg/kg ketamine.

After the intratumoural injection of 0.2 mL of ^166^Ho-MS suspensions in five rabbits, a CT scan was performed according to the clinical protocol described below. Only in rabbit number 5, a higher amount of activity (57.9 MBq) was injected for comparison to quantitative SPECT imaging and depositions outside the tumour. CT and SPECT data were compared to the injected mg Ho, which was determined by the injected activity as described above.

### Patients

Human patients previously treated with direct intratumoural injections of ^166^Ho-MS were analysed to provide an example of clinical CT quantification method. Between 2015 and 2017, four patients with head and neck cancer were referred by their head and neck oncologist. Three patients were treated in a palliative setting. If no other palliative treatment options were available, and nonetheless a strong wish for treatment existed, patients were amenable for direct intratumoural injections of ^166^Ho-MS, with the aim of improving the patients’ quality of life. One patient was part of a prospective clinical pilot study (NCT02975739) [8]. All patients provided informed consent before treatment. Immediately after two to four ultrasound-guided intratumoural injections, containing a total of 100 mg of ^166^Ho-MS, a SPECT, a high-dose CT, and a planar scintigraphy of thorax and abdomen (imaging time 300) were performed. This retrospective analysis of patients treated in a palliative setting was approved by the medical ethical committee of the University Medical Center Utrecht. Some study subjects or cohorts have been previously reported [8].

### CT and SPECT

All CT and SPECT imaging was performed using a Symbia T16 SPECT/CT system (Siemens, Erlangen, Germany) that combines a dual-headed gamma camera with a 16-slice CT system. The acquisition parameters were identical to a diagnostic high-dose CT scan of the head and neck region in the clinical setting: tube voltage 110 kVp, effective tube current 225 mAs; detector configuration 16 × 0.6 mm; rotation time 0.6 s; helical scan mode; pitch 1.0. Images were reconstructed with a 1.5-mm slice thickness with a 0.7 mm increment (voxels size 0.56 × 0.56 × 0.7 mm) and a B31s medium smooth reconstruction kernel.

Medium-energy low-penetration collimators were used on both SPECT cameras. Energy windows were set at 80.6 keV (15% window width) for the ^166^Ho photopeak. A total of 120 projections of 30 s were acquired in a 360° noncircular orbit. Quantitative image data were reconstructed to a 128^3^ matrix with an isotropic voxel size of 4.8 mm^3^. The reconstructions were performed using previously validated Monte-Carlo-based reconstruction software [2, 16] using an ordered subsets expectation maximisation algorithm (10 iterations with 8 subsets) and a quantitatively correct forward model, resulting in an absolute quantitative three-dimensional activity distribution in MBq/voxel [2]. Based on the recovered activity and the specific activity of the microspheres, the absolute amount of Ho (mg) was calculated.

### Data analysis

CT data were analysed with ImageJ version 1.50b (NIH, Bethesda, USA). In the phantom of both HoCl_3_ and Ho-MS, a circular region of interest (ROI) with a 10-mm diameter was drawn in the centre of the 5-mL Eppendorf tube. This ROI was applied over 30 slices to create a volume of interest of 1.6 cm^3^. Subsequently, HU value of this VOI (mean ± standard deviation [SD]) was calculated. A scatterplot of the observed HU values against the calculated concentration values was made to obtain a calibration curve.

In the chicken muscle tissue experiments, an VOI was drawn in an area without microspheres. From this VOI, the mean ± SD and maximum HU values were obtained. In the animals and patients, the tumour volume was manually segmented on CT images by one of the investigators (RCB). Based on the literature, it was assumed that all tumour voxels had a HUvalue between -50 and 100 [17] and that all voxels with a HU > 100 contained Ho microspheres. The amount of Ho was calculated using the following strategy:

1. All voxels with HU > 100 were selected and divided by the HU/concentration calibration curve, to obtain a concentration of mg/ml/voxel;
2. This concentration was multiplied by the voxel volume (0.22 mm^3^) to calculate the absolute amount of Ho per voxel;
3. The total sum of these voxels resulted in the total amount of Ho in milligrams.

Continuous data were presented as mean ± SD deviation if normally distributed and as the median and range if skewed (Shapiro-Wilk test). A Pearson correlation coefficient between the dose calibrator, CT-, and SPECT-quantification was calculated if normally distributed, with Spearman rank-order correlation if skewed. Agreement between the measurements was presented as the recovered percentage on SPECT or CT compared to the dose calibrator or SPECT. SPSS software (SPSS for Windows, version 22.0; SPSS Inc., Armonk, USA) was used for all analysis.

## Results

### Phantom calibration

**Fig. 1:**
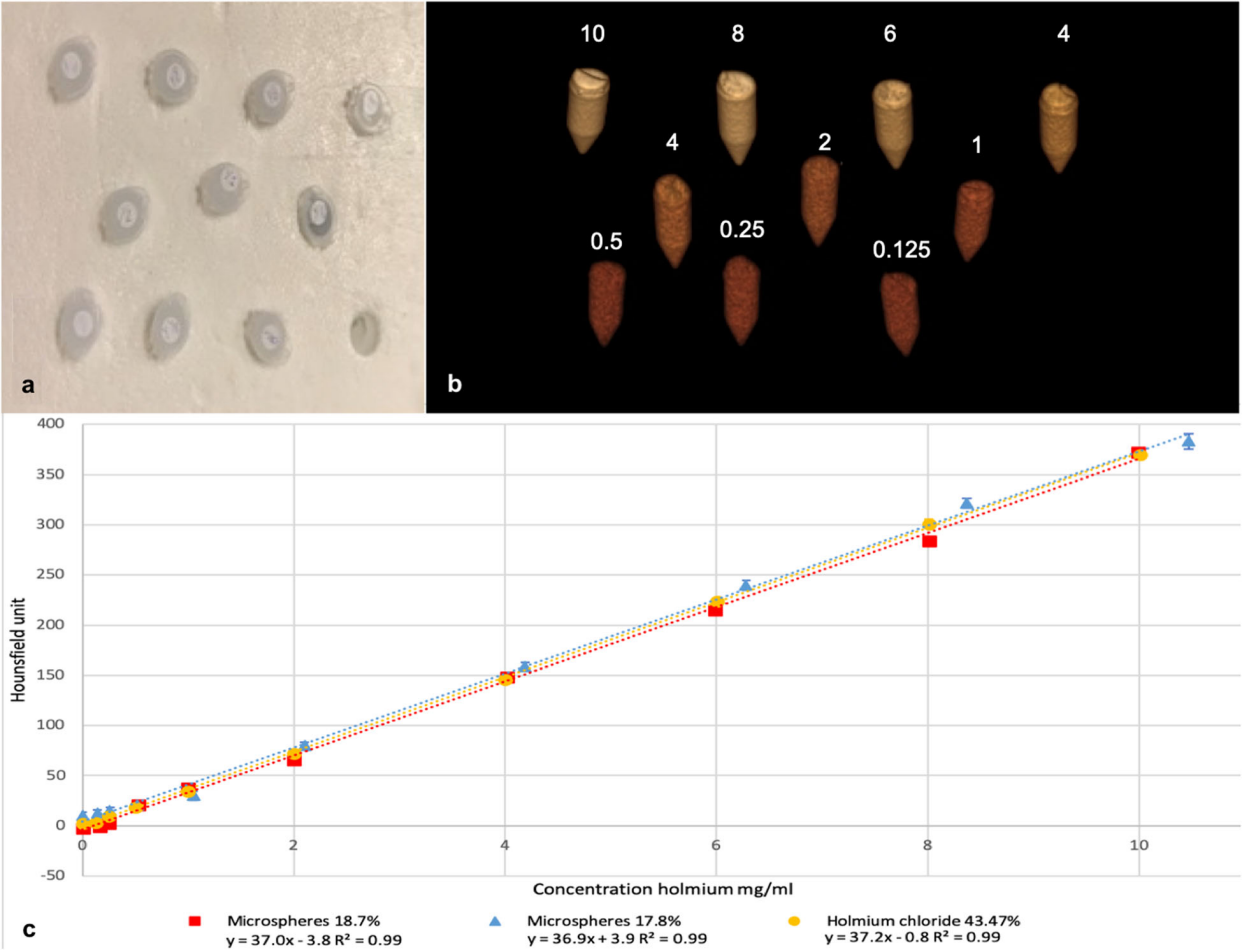
Phantom. **a** The phantom setup of ten 5-mL Eppendorf tubes in styrofoam. **b** Three-dimensional computed tomography reconstruction of the phantom showing the increasing concentrations of Ho in mg/mL. **c** Regression slopes of HU values obtained by the calculated concentration of Ho in mg/mL

The Ho concentration of the ^165^Ho-MS solutions and the HoCl_3_ solution ranged from 0 to approximately 10 mg/mL. Solutions with increasing Ho concentration resulted in increased HU values from around zero HU up to approximately 370 HU (Table 1). The slope of the regression of the three Ho dilution series was 36.9, 37.0, and 37.2 HU per mg/mL of Ho atoms, for the 18.7% and 17.6% ^165^Ho-MS solution and the HoCl_3_ solution, respectively (Fig. 1c). The linear regression between Ho in mg/mL and the HU showed a good fit (least squares *R*^2^ > 0.99) for all series (Fig. 1), without a significant difference between the three lines.

**Table 1:** Phantom Ho quantification

Ho microspheres (18.7%) in agar		Ho microspheres (17.6%) in agar		Ho chloride (43.47%) in water	
Ho	HU	Ho	HU	Ho	HU
mg/mL	Mean ± SD	mg/mL	Mean ± SD	mg/mL	Mean ± SD
0.00	-2.6 ± 8.0	0.00	10.1 ± 7.5	0.00	1.9 ± 6.6
0.16	-0.7 ± 9.9	0.14	12.5 ± 7.1	0.13	3.4 ± 6.5
0.25	2.2 ± 6.9	0.26	15.3 ± 6.1	0.25	9.5 ± 5.8
0.53	20.8 ± 6.8	0.52	20.9 ± 7.6	0.50	18.4 ± 7.1
1.00	37.3 ± 7.1	1.05	30.3 ± 8.8	1.00	34.1 ± 6.9
2.00	65.9 ± 7.1	2.10	79.9 ± 7.2	2.00	72.2 ± 6.3
4.02	148.2 ± 8.2	4.19	158.4 ± 8.1	4.00	145.7 ± 6.9
5.99	215.3 ± 13.4	6.28	239.7 ± 9.5	6.01	223.4 ± 7.2
8.01	283.9 ± 16.8	8.37	321.4 ± 9.4	8.01	301.1 ± 8.2
9.99	372.4 ± 14.1	10.47	383.1 ± 14.8	10.01	370.0 ± 10.5

Observed HU for the 5-mL Eppendorf tubes with a concentration ranging from approximately 0 to 10 mg/mL ^166^Ho-microspheres (18.7, 17.6%) and Ho chloride (43.47%) in styrofoam (as seen in Fig. 1) measured on a Siemens Symbia T16

The feasibility of CT quantification was tested in *ex vivo* chicken muscle tissue. Based on the literature, a threshold of 100 HU was adopted [17]. The VOIs of the chicken muscle tissue without Ho showed a mean HU of 71.2 ± 7.1 with a maximum observed HU value of 96. The chosen cutoff value of > 100 HU was more than the maximum HU value observed and more than three times the SD of unenhanced chicken muscle tissue.

The validated ^166^Ho-SPECT was tested with 5-mL Eppendorf tubes filled with known activities (dose calibrator) and recovered from 99.5 ± 1.4% (mean ± SD) of the activity. SPECT recovered 94.6 ± 4.9% (mean ± SD) of the injected activity in the tissue (Table 2). There was a strong positive correlation between the injected activity and the activity recovered by SPECT (*n* = 5, *r* = 0.99, *p* < 0.001), with a recovered percentage ranging from 90.6 and 101.3%.

**Table 2:** E*x vivo* Ho recovery by single-photon emission computed tomography (SPECT) and computed tomography (CT)

	Injected Ho		SPECT recovery		CT recovery	
	MBq	mg	MBq	%	mg	%
Tissue 1	81.1	18.0	75.9	93.6	16.1	89.8
Tissue 2	73.6	16.3	67.6	91.9	13.2	81.0
Tissue 3	40.3	8.9	36.5	90.6	7.3	81.7
Tissue 4	24.4	5.4	23.1	94.7	4.4	81.5
Tissue 5	15.4	3.4	15.6	101.3	2.5	73.8

Two 5-mL Eppendorf tubes filled with activity were used for SPECT calibration. Five samples of chicken muscle tissue were injected with radioactive microspheres ranging from 15.4 to 81.1 MBq with the corresponding amount of mg Ho. Based on the dose calibrator measurements, SPECT, and CT imaging recovered 90.6–101.3% and 73.8–89.9%, respectively

After injection, the total volume of voxels with a > 100 HU varied from 0.3 to 1.5 cm^3^. A positive correlation was observed between the volume and the amount of injected Ho (*n* = 5, *r* = 0.90, *p* = 0.043). Using the simple method described above, 81.5 ± 6.7% (mean ± SD) of the Ho was recovered using CT (Table 2).

### Laboratory animals

**Fig. 2:**
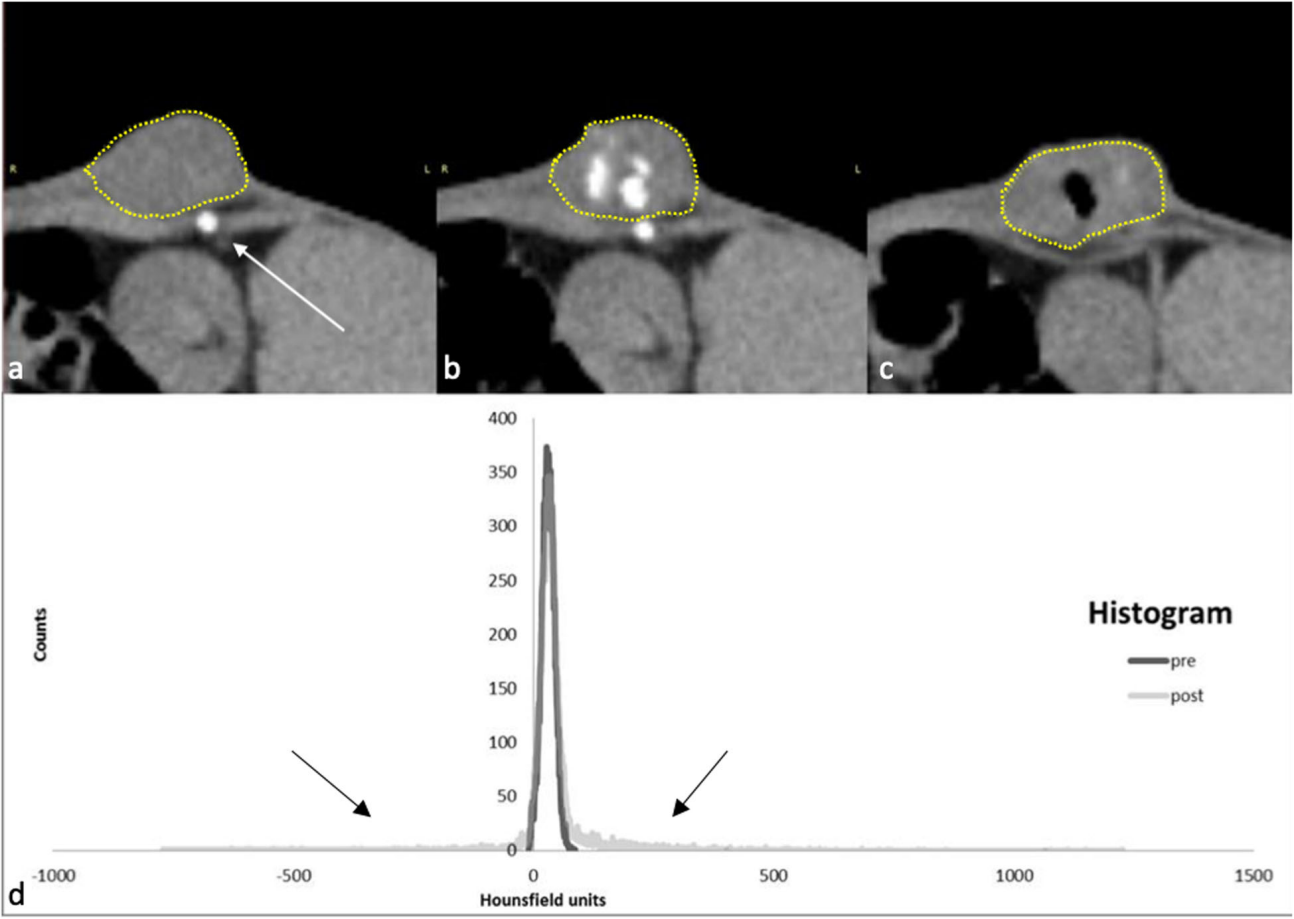
Ho quantification in a subcutaneous VX-2 bearing rabbit. Rabbit 2. **a** Tumour before injection. The tumour is segmented with a yellow dotted line. The white arrow shows a rib. The rib is also visible in **b**. **b** Tumour after injection with accumulations of Ho microspheres, visible as white dots, indicated with white arrows. **c** A different axial slice shows the effect of inadvertent injection of air (black void inside the tumour). **d** Histogram of HU values of the entire tumour before and after injection of ^166^Ho microspheres. The large negative (left of peak) and positive tail (right of peak) are caused by a relatively small number of voxels with injected air or Ho, respectively

Nineteen days after VX-2-tumour implantation, five tumours reached a diameter of approximately 20 mm in the rabbits (Table 3). Before the injection of ^166^Ho-MS, the tumour was segmented on CT (Fig. 2a). The mean tumour volume was 4.8 ± 1.9 cm^3^ (range 3.2–7.2 cm^3^). The mean tumour HU was 31.6 ± 14.3 with a maximum of 98 HU.

**Table 3:** *In vivo* Ho recovery in subcutaneous VX-2 tumour-bearing rabbits by single-photon emission computed tomography (SPECT) and computed tomography (CT)

Rabbit	Injected Ho		Tumour volume	Volume of voxels with HU > 100		CT recovery		SPECT recovery	
	MBq	mg	cm^3^	cm^3^	%	mg	%	MBq	%
1	0.10	3.6	6.6	0.5	6.9	2.8	77.7	NP	
2	0.07	2.2	6.0	0.2	3.5	1.3	59.9*	NP	
3	0.04	1.4	7.2	0.2	3.2	1.1	76.9	NP	
4	0.01	0.4	3.2	0.1	2.5	0.4	100.0	NP	
5	57.9	2.9	3.4	0.3	9.1	1.9	65.4*	54.3	93.8%

Five rabbits with subcutaneous VX-2 tumours were injected with decayed or radioactive microspheres with the corresponding amounts of Ho. The tumour volume ranged from 3.2 to 7.2 cm^3^, based on the HU < 100 threshold while 0.1–0.5 cm^3^ or 2.5–9.1% of the tumour volume was filled with holmium after treatment. Ho recovery by CT ranged from 59.9 to 100.0% (*n* = 5), recovery by SPECT was 93.8% (*n* = 1)

*NP* Not performed

*Evident collections of air on CT resulting in decreased recovery of holmium due to partial volume effects and voxels with large negative HU

After the injection of ^166^Ho-MS, areas with high attenuation were seen inside the tumour caused by the accumulation of microspheres (Fig. 2b). Analysis of the observed HU values in the VOI before and after injection shows a right-skewed distribution due to voxels containing Ho. Accidental injection of air into rabbit 2 and 5 resulted in voxels with highly negative HU (*e.g.*, -722 HU) observed as radiolucent area (Fig. 2c) and a left-skewed distribution (Fig. 2d). The volume of voxels showing > 100 HU values ranged 0.1–0.5 cm^3^ or 3–9% of the total tumour volume.

In these rabbits, 76.0 ± 15.4% (range 59.9–100.0%) of the administered Ho according to the dose calibrator measurements was recovered on CT (Table 3). A positive correlation between the injected amount and the recovered amount on CT was found (*n* = 5, *r* = 0.97, *p* = 0.027) with a recovered percentage ranging from 59.9% to 100%. The SPECT analysis of rabbit 5, injected with 57.9 MBq of ^166^Ho-MS, showed a clear hotspot at the tumour location. Depositions of activity were not seen outside the tumour (*e.g.*, in the kidney or lung). The recovered activity on SPECT was 54.3 MBq, equal to 93.7% of the injected activity.

### Patients

Four patients were treated with intratumoural ^166^Ho-MS injections (Table 4). One patient did not tolerate SPECT imaging due to orthopnoea and was not included in the analysis. Patient 2 was treated on both sides of the neck. The tumour volume of patients ranged from 3.9 to 44.6 cm^3^ (Table 4). According to the dose calibrator, the injected activity ranged from 17.6 to 366.7 MBq, corresponding with an absorbed dose of 111 to 165 Gy. The total volume of voxels with > 100 HU values ranged from 0.5 to 2.7 cm^3^ or 6.1% to 23.1% of the tumour volume.

**Table 4:** Ho recovery in patients single-photon emission computed tomography (SPECT) and computed tomography (CT)

Patient	Tumour volume	Injected Ho		SPECT recovery		CT recovery		Volume of voxels with HU > 100	
	cm^3^	MBq	mg	MBq	%	mg	%	cm^3^	%
1	44.6	366.7	36.5	309.2	84.3	32.4	88.8	2.7	6.1
2 left	5.6	53.9	13.7	9.6	17.8	2.3	16.5	0.5	8.0
2 right	6.1	63.8	16.2	16.9	26.5	3.8	23.5	0.6	9.3
3	3.9	17.6	3.1	8.5	48.3	1.5	48.6	0.9	23.1

Three patients with recurrent head and neck cancer were treated with radioactive ^166^Ho-microspheres and underwent SPECT/CT imaging. Patient 2 was treated on both sides of the neck. Base on the injected activity, the corresponding amount of mg Ho was calculated. Injected activity compared to SPECT recovery was 17.8–84.3%. CT recovery was in line with SPECT imaging ranging from 16.5 to 88.8%. Tumour volume was 3.9–44.6 cm^3^ based on the > 100 HU threshold; from 6.1 to 23.1% of tumour volume was filled with ^166^Ho-microspheres

**Fig. 3:**
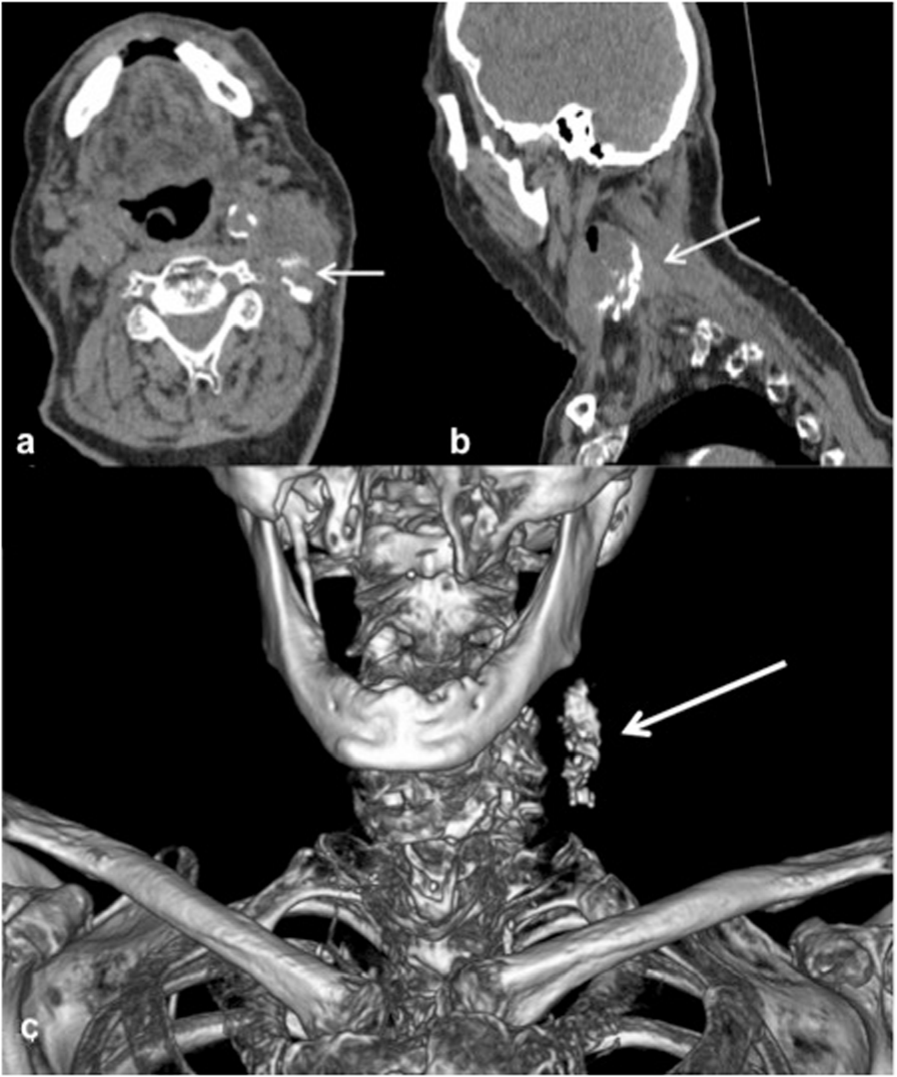
Computed tomography (CT) images of patient number 1. Axial, sagittal, and three-dimensional CT reconstructions of patient number 1 with a large necrotic tumour on the left neck side. During injection, the holmium microspheres were clearly visible on ultrasound as a cloud in the necrotic fluid and did precipitate after some minutes at the bottom of the tumour. The dorsal and caudal accumulation of ^166^Ho microspheres in the tumour is also well visible in the three images obtained in supine position (arrows in **a**, **b**, and **c**)

**Fig. 4:**
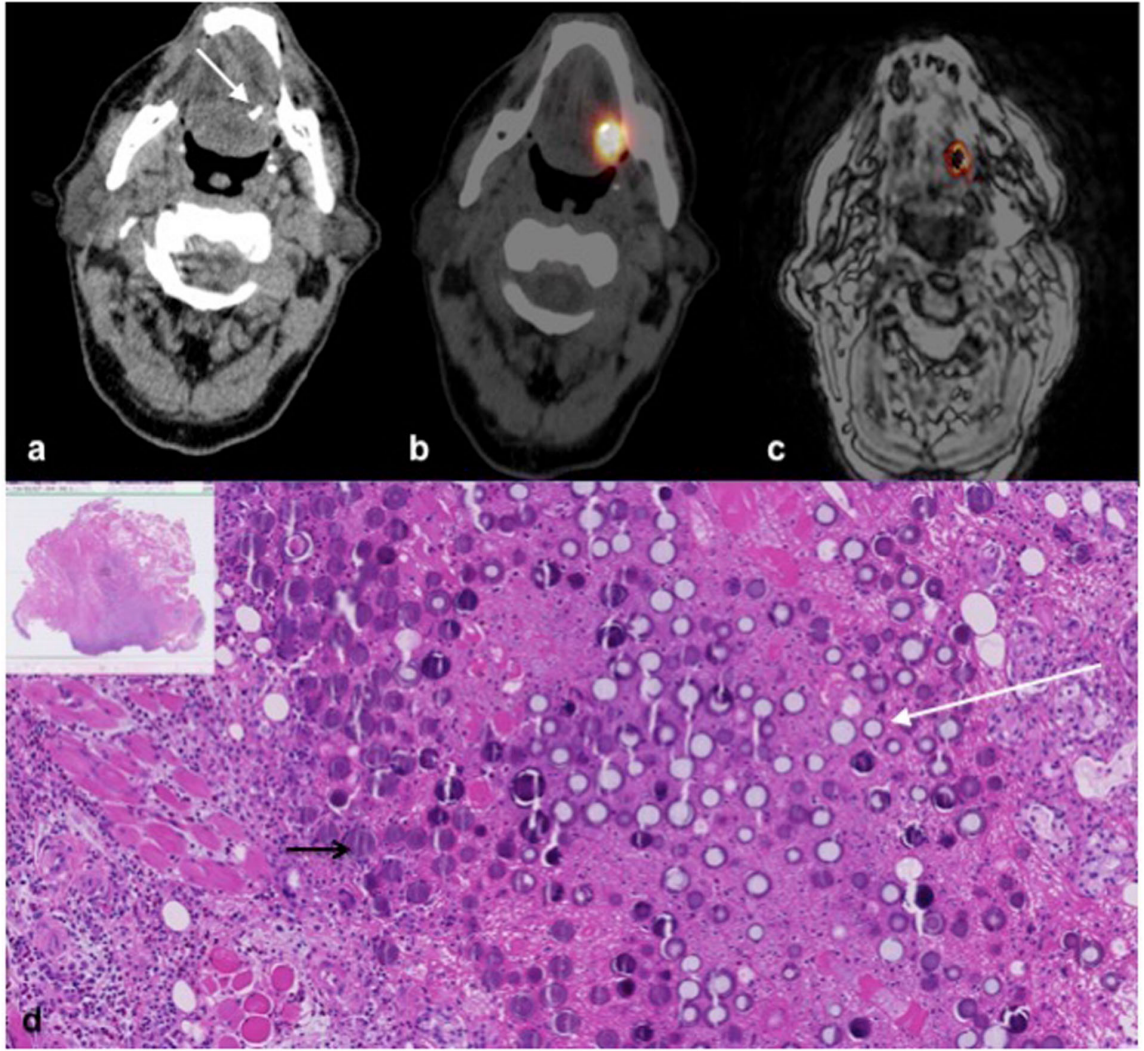
Multimodality imaging and histopathology after injection of ^166^Ho microspheres in patient number 3. **a** Unenhanced computed tomography (CT); microspheres are visible as hyperdense area on the left side of the tongue (arrow). **b** Single-photon computed tomography/CT; the location of the microspheres dose distribution is observed as a large hotspot. **c** Magnetic resonance imaging; with dose reconstruction derived and overlaid on this image, obtained with a T2* multi-gradient echo weighted sequence acquiring 16 echoes (TR/TE1/ΔTE: 1000 ms/1.33 ms/1.15 ms; flip angle 70°), the black centre has ^166^Ho microsphere concentration > 10 mg/ml, resulting in a rapid signal loss restricting the nonlinear least squares (exponential) fit to compute a T2* value and thus a dose value. **d** Histopathology. The haematoxylin and eosin staining shows a moderately differentiated and partly invasive growing oral squamous cell carcinoma with clusters of ^166^Ho microspheres. The black arrow indicates purple spherical structures that are sliced microspheres. The white arrow indicates white spherical structures that are partly or totally removed microspheres by slicing the tissue in 4-μm slices. In the direct environment of the microspheres, a necrotic tissue is seen, while the periphery is unaffected by radiation. Infiltration of lymphocytes is most likely radiation unrelated and often seen in oral squamous cell carcinoma [18]

In patient number 1, ultrasound guidance showed precipitation of microspheres after injection indicating liquefaction of the tumour centre probably due to tumour necrosis. CT provided a clear distribution and accumulation of the ^166^Ho-MS after injection as shown in Fig. 3. Compared to SPECT and MRI, the deposition of high concentrations of Ho was more accurately depicted on CT. CT could discriminate two separate distributions which merged on SPECT and MRI and was able to quantify higher concentrations than MRI (Fig. 4). The injected ^166^HoMS resulted in local cell death microscopically (Fig. 4).

The CT-based quantification method recovered 88.8% of the injected activity in patient number 1, while 84.3% was recovered by SPECT (Table 4). Recovery of injected activity was much lower for patients number 2 and number 3, probably because of unperceived leakage of activity after intratumoural injection. The CT recovery in all patients corresponded well with SPECT recovery (*ρ* = 1.0, *n* = 4, *p* = 0.045), and CT recovered from 88.7 to 105.3% of the intratumoural activity recovered on SPECT (Table 4).

## Discussion

This is the first report investigating the clinical quantification of ^166^Ho-MS using CT. CT quantification may be of additional value to the currently used imaging modalities such as MRI and SPECT because of its superior spatial resolution, the opportunity to perform real-time imaging, lower costs, and widespread availability. CT scanning is especially suitable for rapid imaging feedback of the ^166^HoMS distribution and to visualise potentially skipped areas during treatment. Based on the CT imaging during treatment, the distribution of ^166^Ho-MS can be optimised either by injecting additional microspheres if a lethal absorbed dose is not reached or by discontinuing further administration if a lethal dose is met or if undesired spread of ^166^Ho-MS is observed. After treatment, a combined SPECT/CT examination, which allows for both imaging of ^166^Ho-MS distribution and recovery of possible leakage outside the treated region, should be performed.

Holmium has a high CT attenuation coefficient due to its high electron density (atomic number 67) and a K-edge (56 keV) close to the mean energy of the photons emitted in CT (63 keV at a tube voltage of 120 kV) [19]. It has previously been shown that lanthanides like Ho have up to 50% higher x-ray attenuation than iodine in CT [20]. The phantoms with HoCl_3_ and two batches of ^166^Ho-MS showed a comparable linear slope of 36.9–37.2 HU/mg/mL of Ho with a good linear fit suggesting that this regression can be used for the entire range of concentrations.

The feasibility of CT-based quantification was initially tested on *ex vivo* muscle tissue. A threshold of 100 HU was used to derive quantification of ^166^Ho-MS. This threshold of 100 HU was selected based on the literature [17] and on the current data in this article. The maximum HU in all *ex vivo* muscle tissues was below 100. In addition, this was more than three times the SD of muscle tissue and segmented VX-2 tumours in rabbits. However, Ho recovery up to 2.7 mg/mL (100 HU/37.0 HU/mg/mL Ho) may be difficult. A lower threshold would lead to the recovery of more Ho but would also result in increased noise, while a higher threshold would result in a higher specificity at the cost of sensitivity. The used > 100 HU threshold remains arguable. Nevertheless, using this simple quantification method, 59.9–100.0% of the injected microspheres were recovered.

There are several advantages of CT-based Ho quantification as compared to the current SPECT imaging. First, a more accurate assessment of the intratumoural distribution of ^166^Ho-MS is possible because of a better spatial resolution of CT. The fact that the β radiation of ^166^Ho has an average penetration range of only a few millimetres while 90% of the dose is absorbed whithin 2.1 mm [21] stresses the importance of using high-resolution imaging. Additional injections, based on the current microsphere distribution, could provide a more homogeneously absorbed dose and subsequently a more favourable treatment outcome. CT imaging can provide such high-resolution imaging in a few seconds, compared with 45 min for SPECT, thus enabling the real-time use of CT for feedback for subsequent injections. Furthermore, in case of intratumoural treatment, relatively high radiation doses (200–800 Gy) [6] are used with higher concentrations of microspheres, resulting in non-quantifiable artefacts on MRI.

The standard of reference for the quantification of ^166^Ho-MS remains SPECT. First, because of the superior sensitivity at low amounts of activity compared with CT as the Ho CT detection limit, under optimal circumstances in a homogeneous solution (0.057 mg/mL) is about one hundred times higher than that of SPECT (0.00054 mg/mL) [22]. Especially in the case of accidental extratumoural deposition, voxels with low Ho concentrations may be not discriminated from normal tissues on CT. Furthermore, SPECT directly quantifies activity, while CT detects only Ho as a metal in terms of electronic density, which is indirectly used to quantify activity by multiplying the amount of microspheres with the known specific activity [22]. On the other hand, SPECT has a significantly lower spatial resolution resulting in a limited sensitivity for intratumoural inhomogeneities and insufficient detail of the actual dose distribution within the treated tumour.

The method presented in this work has several limitations. First, in this feasibility study, there was a limited number of images available for analysis. The Pearson correlation showed a strong correlation, and the recovered percentage was within acceptable range. However, more advanced statistical analyses to assess the absolute agreement were not performed due to the small sample size. Second, the CT acquisition protocols, including tube current, dose, image reconstruction kernels, and equipment, all may have had an impact on CT-based quantification and could be further optimised. Third, the > 100 HU threshold may not be the best threshold in each individual patient and tissue. In addition, tissue with high density, like bone, calcifications, or foreign bodies such as metallic artefacts and beam-hardening artefact could result in quantification errors on CT. In the presented patients, scatter artefacts were present. However, the effect was minimal at the tumour locations that were injected.

A more specific and sensitive method to determine the content of Ho per voxel is desired. Material decomposition with dual-energy and photon-counting CT may provide more specific and accurate quantification and is currently used for iodine CT-based quantification [19, 23]. Combining the imaging opportunities for treatment planning and evaluation with accurate image-guided and robotic-assisted ^166^Ho-MS administration [24, 25] may result in personalised “dose painting” in which the planned dose is injected on the desired location within the tumour. This combination of imaging and robotic administration may ultimately change the treatment paradigm in oncology, particularly for single tumours that may be reached by a needle, including head-neck cancer, brain tumours, and pancreatic tumours that are still associated with a poor prognosis.

In conclusion, we showed the metal Ho in radioactive ^166^Ho-MS for local treatment of tumours can quantified on CT and seems to be a true multimodality-imaging isotope with quantitative imaging capabilities using SPECT, MRI, and CT. CT provides high-resolution imaging of intratumoural ^166^HoMS distribution and enables quantification of high local ^166^HoMS concentrations. CT may especially be suitable for an image-guided treatment approach, providing rapid intraprocedural imaging feedback of the ^166^HoMS distribution, directing subsequent injections in possible skipped areas. Such a “dose painting” approach may greatly optimise tumour dose coverage and subsequent clinical outcome.

## Data Availability

The datasets generated during and/or analysed during the current study are available from the corresponding author on reasonable request.

## Abbreviations

^166^Ho-MS: Radioactive holmium-166 (^166^Ho)-loaded (poly L-lactic acid) microspheres
3D: Three-dimensional
CT: Computed tomography
MRI: Magnetic resonance imaging
ROI: Region of interest
SD: Standard deviation
SPECT: Single-photon emission computed tomography
VOI: Volume of interest
